# Type I Interferon Autoantibodies Correlate With Cellular Immune Alterations in Severe COVID-19

**DOI:** 10.1093/infdis/jiae036

**Published:** 2024-02-29

**Authors:** Benedikt Strunz, Christopher Maucourant, Adi Mehta, Hui Wan, Likun Du, Dan Sun, Puran Chen, Anna Nordlander, Yu Gao, Martin Cornillet, Jonna Bister, Egle Kvedaraite, Wanda Christ, Jonas Klingström, Daniel Geanon, Åsa Parke, Anna Ekwall-Larson, Laura Rivino, Paul A MacAry, Soo Aleman, Marcus Buggert, Hans-Gustaf Ljunggren, Qiang Pan-Hammarström, Fridtjof Lund-Johansen, Kristoffer Strålin, Niklas K Björkström, the Karolinska KI/K COVID-19 Study Group, Anders Sönnerborg, Lena Dillner, Hedvig Glans, Pontus Nauclér, Olav Rooyackers, Johan Mårtensson, Lars I Eriksson, Björn P Persson, Jonathan Grip, Christian Unge, Therese Djärv, Dorota Religa, John Tyler Sandberg, Helena Bergsten, Susanna Brighenti, Marta Butrym, Benedict J Chambers, Angelica Cuapio, Isabel Diaz Lozano, Majda Dzidic, Johanna Emgård, Malin Flodström-Tullberg, Jean-Baptiste Gorin, Alvaro Haroun-Izquierdo, Laura Hertwig, Sadaf Kalsum, Efthymia Kokkinou, Nicole Marquardt, Magdalini Lourda, Kimia T Maleki, Karl-Johan Malmberg, Jakob Michaëlsson, Jenny Mjösberg, Kirsten Moll, Jagadeeswara Rao Muvva, Anna Norrby-Teglund, Laura M Palma Medina, Tiphaine Parrot, Lena Radler, Emma Ringqvist, Johan K Sandberg, Takuya Sekine, Tea Soini, Mattias Svensson, Janne Tynell, Andreas von Kries, David Wullimann, André Perez-Potti, Olga Rivera-Ballesteros, Renata Varnaite, Mira Akber, Lena Berglin, Demi Brownlie, Marco Giulio Loreti, Ebba Sohlberg, Tobias Kammann, Elisabeth Henriksson, Quirin Hammer

**Affiliations:** Center for Infectious Medicine, Department of Medicine Huddinge, Karolinska Institutet, Karolinska University Hospital, Stockholm, Sweden; Center for Infectious Medicine, Department of Medicine Huddinge, Karolinska Institutet, Karolinska University Hospital, Stockholm, Sweden; Department of Immunology, Oslo University Hospital, Oslo, Norway; Department of Biosciences and Nutrition, Karolinska Institutet, Stockholm, Sweden; Department of Biosciences and Nutrition, Karolinska Institutet, Stockholm, Sweden; Center for Infectious Medicine, Department of Medicine Huddinge, Karolinska Institutet, Karolinska University Hospital, Stockholm, Sweden; Center for Infectious Medicine, Department of Medicine Huddinge, Karolinska Institutet, Karolinska University Hospital, Stockholm, Sweden; Department of Cellular Therapy and Allogeneic Stem Cell Transplantation, Karolinska University Hospital Huddinge, Stockholm, Sweden; Center for Infectious Medicine, Department of Medicine Huddinge, Karolinska Institutet, Karolinska University Hospital, Stockholm, Sweden; Center for Infectious Medicine, Department of Medicine Huddinge, Karolinska Institutet, Karolinska University Hospital, Stockholm, Sweden; Center for Infectious Medicine, Department of Medicine Huddinge, Karolinska Institutet, Karolinska University Hospital, Stockholm, Sweden; Center for Infectious Medicine, Department of Medicine Huddinge, Karolinska Institutet, Karolinska University Hospital, Stockholm, Sweden; Department of Pathology and Cancer Diagnostics, Karolinska University Hospital, Stockholm, Sweden; Center for Infectious Medicine, Department of Medicine Huddinge, Karolinska Institutet, Karolinska University Hospital, Stockholm, Sweden; Center for Infectious Medicine, Department of Medicine Huddinge, Karolinska Institutet, Karolinska University Hospital, Stockholm, Sweden; Center for Infectious Medicine, Department of Medicine Huddinge, Karolinska Institutet, Karolinska University Hospital, Stockholm, Sweden; Department of Medicine Huddinge, Karolinska Institutet, Stockholm, Sweden; Department of Infectious Diseases, Karolinska University Hospital, Stockholm, Sweden; Department of Laboratory Medicine, Division of Clinical Microbiology, Karolinska Institutet, Stockholm, Sweden; Programme in Emerging Infectious Diseases, Duke-National University of Singapore Medical School, Singapore, Singapore; School of Cellular and Molecular Medicine, University of Bristol, Bristol, United Kingdom; Department of Microbiology and Immunology, Yong Loo Lin School of Medicine, National University of Singapore, Singapore, Singapore; Department of Infectious Diseases, Karolinska University Hospital, Stockholm, Sweden; Infectious Diseases, Department of Medicine Huddinge, Karolinska Institutet, Stockholm, Sweden; Center for Infectious Medicine, Department of Medicine Huddinge, Karolinska Institutet, Karolinska University Hospital, Stockholm, Sweden; Center for Infectious Medicine, Department of Medicine Huddinge, Karolinska Institutet, Karolinska University Hospital, Stockholm, Sweden; Department of Biosciences and Nutrition, Karolinska Institutet, Stockholm, Sweden; Department of Immunology, Oslo University Hospital, Oslo, Norway; Department of Medicine Huddinge, Karolinska Institutet, Stockholm, Sweden; Department of Infectious Diseases, Karolinska University Hospital, Stockholm, Sweden; Center for Infectious Medicine, Department of Medicine Huddinge, Karolinska Institutet, Karolinska University Hospital, Stockholm, Sweden

**Keywords:** COVID-19, autoantibodies, interferon, immunity

## Abstract

**Background:**

Infection with severe acute respiratory syndrome coronavirus 2 (SARS-CoV-2) can lead to severe disease with increased morbidity and mortality among certain risk groups. The presence of autoantibodies against type I interferons (aIFN-Abs) is one mechanism that contributes to severe coronavirus disease 2019 (COVID-19).

**Methods:**

This study aimed to investigate the presence of aIFN-Abs in relation to the soluble proteome, circulating immune cell numbers, and cellular phenotypes, as well as development of adaptive immunity.

**Results:**

aIFN-Abs were more prevalent in critical compared to severe COVID-19 but largely absent in the other viral and bacterial infections studied here. The antibody and T-cell response to SARS-CoV-2 remained largely unaffected by the presence aIFN-Abs. Similarly, the inflammatory response in COVID-19 was comparable in individuals with and without aIFN-Abs. Instead, presence of aIFN-Abs had an impact on cellular immune system composition and skewing of cellular immune pathways.

**Conclusions:**

Our data suggest that aIFN-Abs do not significantly influence development of adaptive immunity but covary with alterations in immune cell numbers.

## Teaser

This study shows that autoantibodies against type I interferon covary with cellular alterations, in particular reduced immune cell numbers, while they have limited effect on the soluble and adaptive immune response in critical COVID-19.

The immune response mediated by type I interferons (IFNs) is important for the control of viral infections. However, this system appears to be out of balance in some patients with severe coronavirus disease 2019 (COVID-19) [1, 2]. Inborn errors in the type I IFN pathway have been associated with critical COVID-19 [3] and other severe viral diseases [4, 5]. Autoantibodies against type I IFN (aIFN-Abs) are present in up to 15% of patients with critical COVID-19 but only rarely in healthy or asymptomatic individuals [6–8]. Their presence was also linked to a higher risk of mortality in COVID-19 [9]. While it has been shown that aIFN-Abs in COVID-19 hamper the downstream type I IFN-stimulated gene (ISG-I) response and, to some extent, disturb the cellular immune composition [7], little is known about their impact on the soluble immune compartment and their impact on humoral immunity. Together, these findings highlight the importance of a functional type I IFN response system in infection with severe acute respiratory syndrome coronavirus-2 (SARS-CoV-2) and suggest that perturbations in this system, such as via the presence of aIFN-Abs, associate with a more severe course of disease.

The type I IFN family consists of several subtypes, including IFN-α, IFN-β, IFN-ω, and others [10, 11]. IFNs are known to participate in local antiviral defense reactions but also function on a systemic level affecting both immune cells and soluble immune factors [11, 12]. Of interest, aIFN-Abs are mostly directed against IFN-α and are capable of neutralizing the targeted type I IFNs [6, 13]. Beyond COVID-19, the presence of aIFN-Abs has been associated with increasing age. In this respect, 4% of individuals older than 70 years harbor such antibodies [13]. Mutations in the type I IFN pathway also associate with other severe viral infections [4, 14]. While there are reports on the association of aIFN-Abs with other conditions, such as adverse reactions following yellow fever vaccination [15], severe varicella-zoster infection [16], West Nile virus infection [17], and influenza pneumonia [18], it is not known if these autoantibodies underlie other common critical infections.

Here, we examined the prevalence of aIFN-Abs in a cohort of patients with severe and critical COVID-19. A particular emphasis is devoted to the temporal dynamics of aIFN-Abs and how the presence of these autoantibodies affects humoral, cellular, and inflammatory immune responses. The results show a predominant effect on the cellular level with largely unaltered soluble immune factors and provide a deeper understanding of the effects of aIFN-Abs on the immune response.

## METHODS

### Study Overview and Subjects

In this study a cohort of 269 patients hospitalized with polymerase chain reaction (PCR)-verified COVID-19 were included, as well as 18 healthy controls. All patients and controls were included during 2020 and early 2021, before introduction of vaccination against SARS-CoV-2. For 12 patients no blood samples were acquired and they were therefore excluded. One patient had no further clinical data available and was only included in comparisons of total patients with autoantibodies (aIFNpos) versus without autoantibodies (aIFNneg) without stratification for clinical parameters. Five patients had only been sampled at later stages (convalescent phase) and were only included for comparison of clinical parameters and antibody analysis. Two sites at Karolinska Hospital were involved in sample acquisition. At the first site, serum and whole blood were collected, a fresh staining on absolute cell counts (Trucount) was conducted, and peripheral blood mononuclear cells (PBMCs) and serum samples frozen. The second site only handled serum samples. In total, serum samples (any time point) were available for a total of 257 patients and PBMC and cell counts for 188 patients. Three samples were excluded from analysis for absolute cell counts, because of either low cell viability or unreliable cell discrimination. Whenever the acute setting was to be compared, patients with a first sampling time point after treatment in the intensive care unit (ICU) were excluded (total n = 17) as we argue that these patients were already progressing towards convalescent phase. When studying the serological response against other viruses all patients were included. Of all included patients, 11 patients tested positive for autoantibodies against at least 1 of the examined IFN subtypes. Eight patients with aIFN-Abs were matched for age, sex, and days from symptom debut to sampling to aIFNneg patients and analyzed with flow cytometry for B- and T-cell phenotype. Patients with hantavirus infection (Puumala virus) were sampled during the acute phase of hemorrhagic fever with renal syndrome (HFRS) during hospitalization. Yellow fever vaccination samples were collected after vaccination of healthy study participants as described elsewhere [19]. Dengue virus samples were collected from hospitalized patients during the acute stage of dengue fever. Samples from 39 sepsis patients were collected at the time of hospitalization; 2 samples failed analysis for aIFN-Abs and were excluded. Data are depicted for the remaining 37 samples; cause for sepsis is detailed in Supplementary Table 1. For methods on serological analysis, B-cell receptor (BCR) sequencing and statistical analysis, see Supplementary Materials.

### Serum Proteome Analysis With Proximity Extension Assay

Of serum samples, 40 µL were aliquoted to skirted PCR plates, frozen, and finally analyzed via proximity extension assay with the Olink Explore 1536 platform consisting of 4 analysis panels (cardiometabolic, inflammation, neurology, and oncology). For proteins measured in more than 1 of the respective panels, only the values from the inflammation panels were included. Additionally, samples and assays failing quality control were excluded, resulting in 1463 proteins to be analyzed. Data was imported in R, groups defined, and differences between those calculated as mean difference of NPX values (random unit in log_2_ transformed format determined by Olink).

### Graphical Display

Schematic figures were created with BioRender.com.

## RESULTS

### Autoantibodies Against Type I IFNs Associate With Severe COVID-19 but Are Largely Absent in Other Infectious Diseases

**Figure 1.**
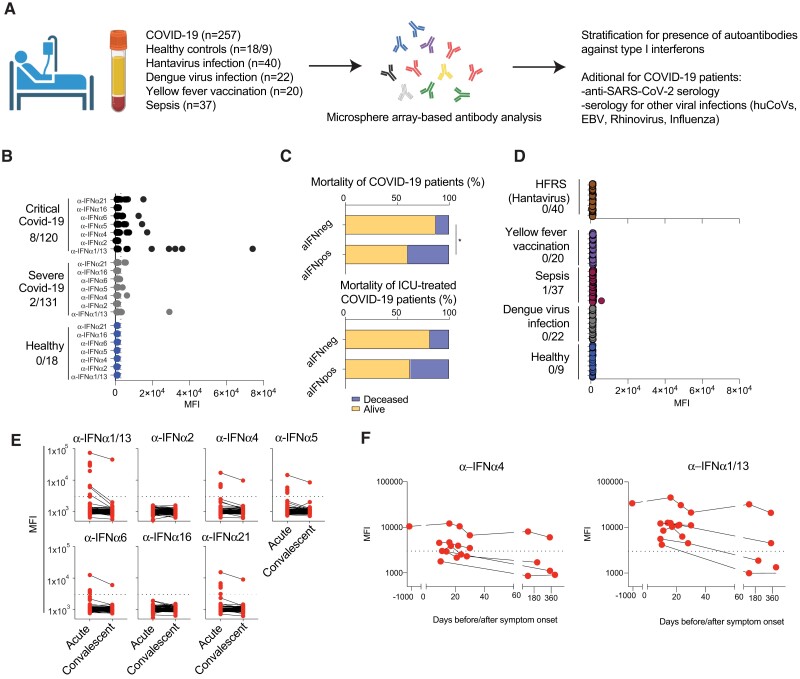
Assessment of aIFN-Abs in COVID-19 and other infections and their impact on antiviral antibody response. *A*, Schematic study design. *B* and *D*, Autoantibodies against the indicated type I IFN subtypes were measured via multiplexed bead assay. Displayed are MFI values in (*B*) healthy controls (n = 18), patients with severe (hospitalized not ICU, n = 131), or critical COVID-19 (ICU patients, n = 120); and (*D*) hantavirus mediated HFRS (n = 40), healthy controls (n = 9), acute dengue virus infection (n = 22), sepsis (n = 37), and yellow fever vaccination at day 7 (n = 20). *C*, Proportion of patients that died or survived COVID-19 infection stratified for positivity for aIFN-Abs; upper plot includes all patients with known outcome (n = 256), lower plot is restricted to ICU patients (n = 121). Fisher exact test was used to determine statistical differences among the groups. *P < .05. *E*, MFI of autoantibodies against indicated IFN subtype in COVID-19 patient samples from the acute (n = 251) or convalescent phase (n = 119). *F*, Analysis of IFN-α4 and IFN-α1/13 as examples of aIFN-Abs in patients with samples acquired before (n = 1, 3 years before) or during the acute phase (n = 7), at convalescent phase (3–6 months follow-up, n = 3), and at 1 year follow-up (n = 3). Abbreviations: aIFN-Abs, IFN autoantibodies; aIFNneg, IFN autoantibodies negative; aIFNpos, IFN autoantibodies positive; COVID-19, coronavirus disease 2019; EBV, Epstein-Barr virus; HFRS, hemorrhagic fever with renal syndrome; huCoV, human coronavirus; ICU, intensive care unit; IFN, interferon; MFI, mean fluorescence intensity; SARS-CoV-2, severe acute respiratory syndrome coronavirus 2.

As a starting point, the presence and dynamics of aIFN-Abs were investigated in a Swedish cohort of COVID-19 patients with severe and critical disease. To this end, the presence of aIFN-Abs against seven IFN-α subtypes was determined in 257 patients that were hospitalized in 2020 and early 2021 (Figure 1*A*). The assay for detection of aIFN-Abs was validated with clinical samples from 2 patients with autoimmune polyendocrine syndrome type I (APS-1) as positive controls (Supplementary Figure 1*A*). In line with previous research [3, 8], aIFN-Abs were observed in COVID-19 but not in healthy controls. In COVID-19, these autoantibodies were more prevalent in patients with critical (ICU-treated patients) as compared to severe disease (Figure 1*B* and Supplementary Table 1). When stratifying the total cohort of COVID-19 patients for presence of aIFN-Abs, we noted higher degrees of mortality (Figure 1*C*) whereas other patient characteristics, including age, sex, comorbidity, and body mass index were comparable between patients with (aIFNpos) and without (aIFNneg) autoantibodies (Supplementary Table 1). Differences in clinical laboratory parameters between the 2 patient groups were largely driven by higher degree of severity in aIFNpos patients (Supplementary Table 1). Given the frequent presence of aIFN-Abs in COVID-19 [13], it was next addressed if these autoantibodies might also associate with other viral infections and/or severe infectious diseases. To this end, the presence of aIFN-Abs was studied in patients with hantavirus-caused HFRS (n = 40), acute dengue virus infection (dengue fever) (n = 22), sepsis (n = 39), as well as at 7 days after live attenuated yellow fever virus vaccination (n = 20). Notably, except for 1 patient with sepsis, none of the other 120 patient samples contained detectable aIFN-Abs (Figure 1*D*). Cause for sepsis was mostly bacterial infection (Supplementary Table 1); however, it should be noted that the sepsis patient that tested positive for aIFN-Abs was diagnosed with influenza virus infection. Finally, we also had the possibility, in a subset of COVID-19 patients with aIFN-Abs, to study the dynamics over time (Figure 1*E* and 1*F*). In 1 patient, aIFN-Abs were present at comparable levels already long before the pandemic (prepandemic sample from 2017; Figure 1*F*). In 6 other patients, longitudinal levels of aIFN-Abs were evaluated for up to 1 year after COVID-19. In these patients, levels dropped off over time, with the exception of 1 patient that had similarly high levels of aIFN-Abs up until 1 year after acute COVID-19 (Figure 1*E* and 1*F*). Taken together, we confirm previous reports demonstrating that the presence of aIFN-Abs is associated with severe COVID-19. In contrast, we could not detect the autoantibodies in the other infections tested here.

### Impact of Type I IFN Autoantibodies on the Soluble Inflammatory Response

**Figure 2.**
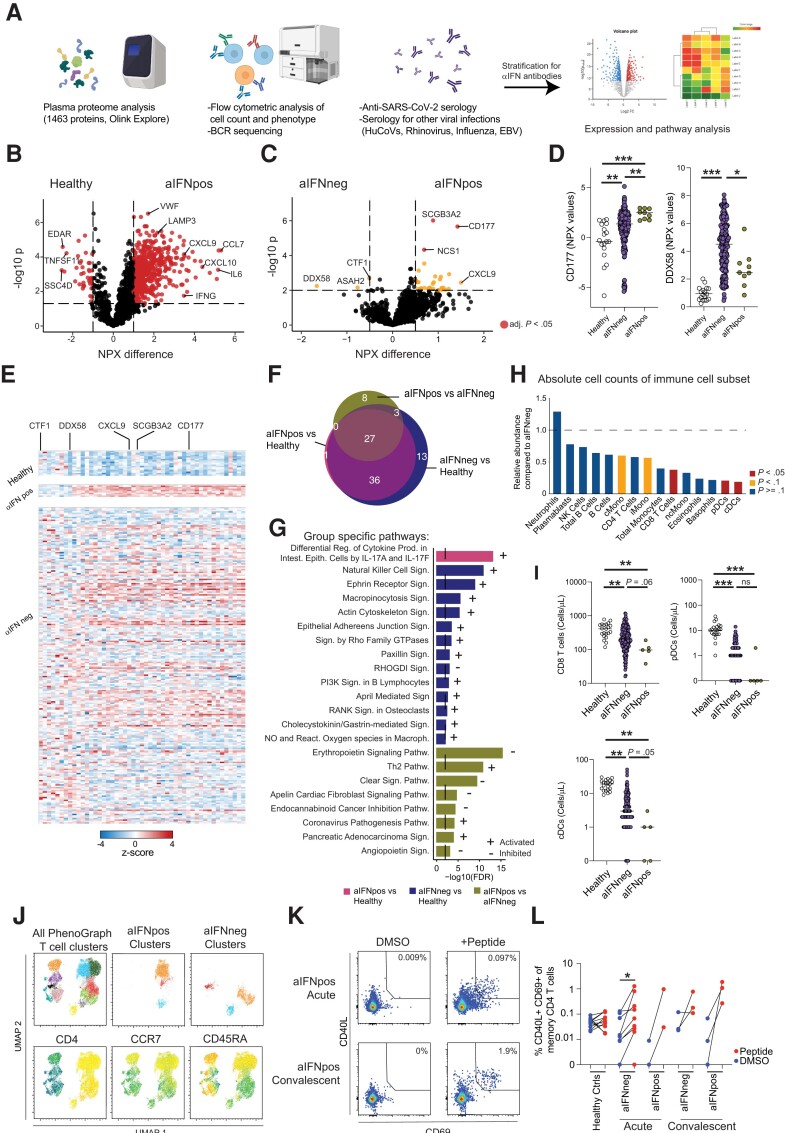
Autoantibodies against type 1 IFN modulate the cellular immune compartment. *A*, Schematic of performed analysis. *B* and *C*, Volcano plot of soluble proteome (1463 proteins) analyzed with proximity extension analysis (OLINK Explore panel) of healthy controls (n = 18) or COVID-19 patients at the first time point of sampling during acute disease stratified for absence (n = 225, aIFNneg) or presence (n = 9, aIFNpos) of autoantibodies against IFN: (*B*) comparison of healthy controls and aIFNpos individuals; and (*C*) aIFNpos and aIFNneg patients. Samples were tested for significant differences with *t* test and false discovery rate-adjusted *P* values for multiple comparison, displayed are unadjusted *P* values in yellow for *P*<0.05 and in red adjusted *P* values <.05. *D*, Exemple plot displaying raw NPX values for CD177 and DDX58; differences among the groups were calculated with Kruskal-Wallis test followed by Dunn correction for multiple comparison. *E*, Heatmap displaying z scores of the differentially expressed genes between aIFNpos and aIFNneg patients, as in (*C*), for healthy controls, and aIFNpos and aIFNneg patients. *F*, Venn diagram displaying shared and distinct pathways calculated with the IPA for the different comparisons of the 3 groups. *G*, Overview of the specific IPA pathways in (*F*) for the indicated comparisons. *H*, Ratio of average absolute lymphocyte counts when comparing aIFNpos (n = 5) to aIFNneg (n = 174) patients in the acute phase of COVID-19. Groups were compared with Mann-Whitney test; blue *P* > = .1, yellow *P* < .1, red *P* < .05. *I*, Absolute counts of CD8 T cells, pDC, and cDC for healthy controls (n = 10), and aIFNneg (n = 174) and aIFNpos COVID-19 patients (n = 5). *J*, UMAP and PhenoGraph clustering analysis from flow cytometric phenotyping of T cells. Included were aIFNpos and aIFNneg donors (each n = 7) at first time point of sampling that were concatenated and split according to aIFN positivity after the respective analysis. To determine the most frequent clusters in aIFNneg and aIFNpos individuals the relative contribution to the cluster was calculated (see also Supplementary Figure 4). *K* and *L*, T-cell function in healthy controls (n = 11), and aIFNneg (n = 9) and aIFNpos patients (n = 5) upon DMSO or SARS-CoV-2 peptide stimulation. *P < .05, **P < .01, ***P < .001. Abbreviations: aIFNneg, IFN autoantibodies negative; aIFNpos, IFN autoantibodies positive; UMAP, uniform manifold approximation and projection; BCR, B cell receptor; cDC, conventional dendritic cells; COVID-19, coronavirus disease 2019; DMSO, dimethyl sulfoxide; EBV, Epstein-Barr virus; huCoV, human coronavirus; IFN, interferon; IPA, ingenuity pathway analysis; ns, not significant; pDC, plasmocytoid dendritic cells; SARS-CoV-2, severe acute respiratory syndrome coronavirus 2.

To investigate possible alterations in soluble immune factors in patients with autoantibodies to type I IFNs, the soluble proteome was examined in healthy controls, aIFNpos, and aIFNneg patients at the acute stage of COVID-19 by assessing 1463 proteins via a proximity extension assay (Figure 2*A*). First we compared the soluble proteome from aIFNpos COVID-19 patients to healthy individuals and observed a significant inflammatory response in aIFNpos patients with drastically elevated levels of proteins such as interleukin 6 (IL-6), IFN-γ, CXCL10, and CCL7 (Figure 2*B*). This pattern was comparable to the inflammatory response seen in aIFNneg COVID-19 patients (Supplementary Figure 2*B*). Given that aIFNpos patients also mounted a significant inflammatory response, it was not surprising that only a few differences were detected in a direct comparison of aIFNpos and aIFNneg patients. In detail, levels of CD177, a marker for neutrophil activation, and CXCL9 were elevated, while DDX58, also known as RIG-I, a sensor for a viral infection that induces type I IFN production [20], was found to be decreased in aIFNpos patients (Figure 2*C*–*E*). Similar findings were observed when studying only ICU-treated patients stratified for aIFN-Ab positivity, apart from DDX58 that might be more related to disease severity in general rather than aIFN-Abs (Supplementary Figure 1*B*–*D*). Next, shared and specific pathways based on the proteome data were investigated in the total cohort of aIFNpos and aIFNneg patients as well as healthy individuals (Figure 2*F* and 2*G*). Whilst the majority of identified pathways were shared between the 2 groups of patients, aIFNpos patients were specifically enriched for Th2 and “coronavirus pathology” pathways, while aIFNneg patients showed enrichment for natural killer cell signaling (Figure 2*F* and 2*G*). Of note, when compiling an ISG score, no difference was noted in aIFNpos compared to aIFNneg patients and equally elevated levels of IFN-λ1 was observed (Supplementary Figure 1*E*).

Altogether, these findings suggest that aIFNpos patients can still mount a substantial inflammatory response but display signs of compromised viral control in combination with altered immune activation pathways.

### Autoantibodies Against Type I Interferons Alter the Cellular Immune Profile

The observed alterations in deduced immune pathways from the soluble proteome analyses led us to further investigate the extent to which aIFN-Abs affect the cellular immune compartment. First, absolute immune cell counts were determined in fresh whole blood from all patients at the acute stage of COVID-19 (Figure 2*A*). A general reduction in absolute immune cell counts was observed in aIFNpos patients in comparison to aIFNneg patients (Figure 2*H*). More specifically, a significant reduction of CD8 T cells as well as of conventional dendritic cells (DCs) and, to some extent also, plasmacytoid DCs was observed, both when investigating the total cohort and when only assessing patients with severe COVID-19 (ICU patients) stratified for aIFN-Ab positivity (Figure 2*H* and 2*I*, and Supplementary Figure 1*E* and 1*F*). Because T cells showed reduced cell counts and enrichment of T helper pathways in our soluble proteome analysis, we next performed 28-color flow cytometry phenotyping and functional analysis of the T-cell compartment in patients with or without aIFN-Abs. Here, we compared 8 aIFNpos patients that were matched for age, sex, disease severity, and time since symptom debut with corresponding aIFNneg patients. Surprisingly, with respect to the T-cell phenotype, no major differences were observed (Figure 2*J*, Supplementary Figure 2, and Supplementary Figure 3*A*). Uniform manifold approximation and projection (UMAP) and PhenoGraph analysis revealed enrichment for naive CD4 T cells and a slight reduction in memory CD4 T cells in aIFNpos patients, but no significant differences were evident in a direct comparison of these 2 groups of patients (Figure 2*J*, Supplementary Figure 2, and Supplementary Figure 3*A*). In some of the patients, T-cell function could additionally be assessed. T cells from aIFNpos patients displayed robust peptide-specific functionality both at the acute and convalescent phases (Figure 2*K* and 2*L*).

These data suggest that there is a predominant loss of immune cells in the periphery of patients with aIFN-Abs, while the T-cell phenotype and function of remaining cells remained largely unaltered.

### Intact Humoral Immunity Despite Type I IFN Autoantibodies

**Figure 3.**
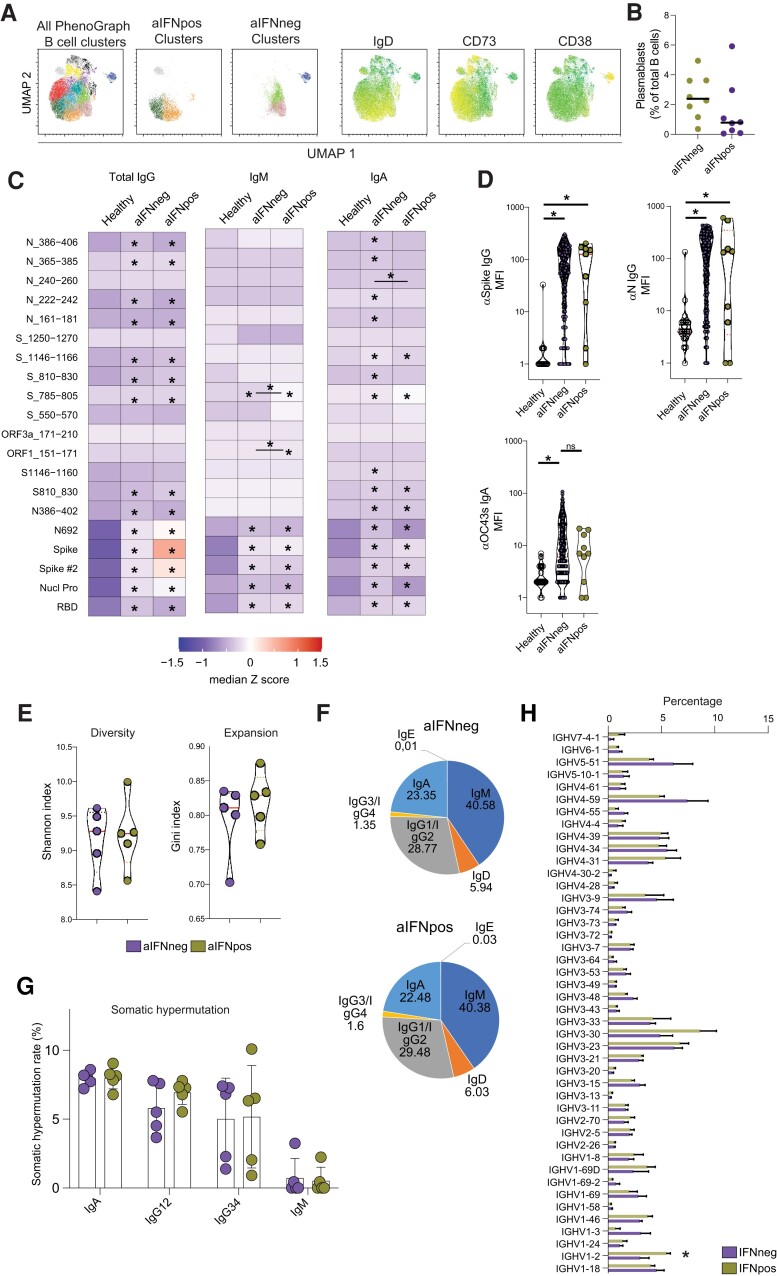
Intact humoral immunity despite type I IFN autoantibodies. *A* and *B*, Flow cytometric analysis of the B-cell compartment. Displayed are UMAP and PhenoGraph analysis for the most enriched clusters (*A*, see also Supplementary Figure 4**)** as well as exemplary summary data (*B*) for aIFNpos and aIFNneg patients (each n = 8). *C* and *D*, Microsphere array-based analysis of antibodies specific against SARS-CoV-2 or other virus proteins or peptides. *C*, Levels of IgG, IgM, and IgA antibodies against the indicated SARS-CoV-2 peptides or full-length proteins. Displayed are median z-scores for healthy (n = 18), and aIFNneg (n = 225) and aIFNpos (n = 9) patients during acute stage of COVID-19. *D*, Exemple data of IgG antibody levels against SARS-CoV-2 and IgA response against spike protein from the seasonal cold coronavirus OC43 (n = 18 healthy, n = 241 aIFNneg, and n = 10 aIFNpos patients). *E–H*, Results from B-cell receptor sequencing from aIFNpos and matched aIFNneg patients during acute disease (each n = 5). Displayed are diversity and expansion indices (*E*), antibody isotype composition (*F*), somatic hypermutation rate (*G*), and IGHV preference (*H*). The median of somatic hypermutation rate and IGHV frequency were calculated for each sample. *C* and *D*, significant differences were tested with Kruskal-Wallis test followed by Dunn test for multiple comparisons. *E–G*, Differences were calculated by Mann-Whitney test. *P < .05. Abbreviations: aIFNneg, IFN autoantibodies negative; aIFNpos, IFN autoantibodies positive; UMAP, uniform manifold approximation and projection; COVID-19, coronavirus disease 2019; IFN, interferon; Ig, immunoglobulin; MFI, mean fluorescence intensity; ns, not significant; SARS-CoV-2, severe acute respiratory syndrome coronavirus 2.

It is known that type I IFNs influence B cells and antibody production [10, 21]. Therefore, we performed flow cytometric phenotyping of B cells, investigated specific Ab responses, and assessed the BCR repertoire in relation to aIFN-Abs. First, the composition of the B-cell compartment and subset phenotypes were examined. No significant differences were noted beyond the observation of lower levels of plasmablasts in some of the aIFNpos patients (Figure 3*A* and 3*B*, Supplementary Figure 3*B*, and Supplementary Figure 4). Next, we assessed if aIFN-Abs altered antibody responses against SARS-CoV-2 and other common viral infections. aIFNpos patients were able to mount a significant anti-SARS-CoV-2 response in the acute phase of COVID-19, not different from aIFNneg patients (Figure 3*C* and 3*D*, and Supplementary Figure 5*A*). When investigating the antibody response to other viral infections, including influenza, EBV, and rhinovirus, generally lower levels of Abs were observed towards these viruses in both aIFNpos and aIFNneg patients than in healthy controls (Supplementary Figure 5*B* and 5*C*). In contrast, Ab production against seasonal coronaviruses appeared elevated, and levels of anti-spike IgG for the strain OC43 were significantly elevated in both aIFNpos and negative patients when compared to healthy controls (Figure 3*C* and Supplementary Figure 5*B*). Finally, we also investigated the BCR repertoire in aIFNpos and negative patients. Only minor differences were found within the BCR repertoire of aIFNpos patients. No significant changes in the overall BCR diversity, clonal expansion isotype composition, and somatic hypermutation could be found (Figure 3*E*–*G*). Only a preference for IGHV1–2 could be observed in aIFNpos patients (Figure 3*H*).

Taken together, these data show that the presence of aIFN-Abs does not overly affect the BCR repertoire and the Ab response toward SARS-CoV-2 and other viral infections.

## DISCUSSION

In the present study, we examined if autoantibodies against type I IFNs observed in a subset of patients with severe COVID-19 covaried with the composition of the soluble and cellular immune compartment. The aIFN-Abs observed here in a subset of severe COVID-19 patients are in line with previous studies where similar frequencies of aIFN-Abs have been reported [6, 7, 13]. Similarly, the observed temporary elevation of levels of aIFN-Abs during acute COVID-19 is also in line with previous data [22, 23].

While the presence of aIFN-Abs has been associated with varicella zoster [16] and herpesvirus replication during COVID-19 [24], we did not find similar autoantibodies in clinical material from patients with acute dengue fever, HFRS, sepsis, or healthy individuals having undergone yellow fever vaccination. In contrast, a recent report on patients with West Nile virus infection identified that patients with more severe disease harbored aIFN-Abs more frequently [17]. With respect to the role of aIFN-Abs in the other infections studied here, it should be noted that our sample sizes were relatively small and that our patient cohorts of dengue and hantavirus infections are not focused on patients with the most severe disease. Thus, specific associations with the most severe disease stages might not be discovered. In the case of yellow fever vaccination (a live attenuated vaccine), we examined if the vaccination/infection itself could prompt autoantibody production, given the previously reported association of aIFN-Abs with vaccination-related adverse reactions [15]. This appeared not to be not the case, adding further evidence that aIFN-Abs are preexisting instead of being triggered during viral infection. Collectively, it appears that aIFN-Abs are associated with higher risk for more severe disease in certain infections, but further studies are needed to determine if aIFN-Abs represent a more general risk factor in bacterial and viral infections.

It has been previously shown that aIFN-Abs are neutralizing [6, 13], and that the blockade of IFNs can influence peripheral immune cell composition [7]. Therefore, we studied the effect of aIFN-Abs on the soluble immune factors and humoral immunity, and immune cell composition, as well as the B- and T-cell compartment. Addressing immune cell composition at a broader level, we observed significant reduction of several immune cell populations in aIFNpos patients, in particular CD8 T cells and DCs. Relating to these findings, it is worth noting that van der Wijst and collaborators, in their studies of immune cell frequencies, found an imprint of severe COVID-19 as indicated by loss of CD8 T cells and DCs, but no apparent differences between patients with or without autoantibodies [7]. The difference between their results and ours might be explained by the fact that we additionally examined absolute numbers of the immune cells compared to only addressing relative frequencies.

Investigating soluble immune factors, an imprint of the ongoing infection with SARS-CoV-2 on the soluble proteome was found, as also described in earlier studies [25]. In contrast, when comparing all aIFNpos and aIFNneg patients, only a few differences could be observed. For instance, analysis of an ISG-score revealed an induction compared to healthy controls in both aIFNpos and aIFNneg patients possibly suggesting that other IFNs, such as IFN-λ1 may play a more significant role in the event of IFNa neutralization. However, we found DDX58 at lower levels in aIFNpos than aIFNneg patients in the whole cohort, but also at lower levels in aIFNneg patients with critical COVID-19 than those with severe disease. This could indicate that elevated levels of DDX58 implicate better viral control and that DDX58 levels in peripheral blood correlate with the IFN response. In line with this, we observed a skewing of pathways deduced from the soluble proteome towards a Th2 response and coronavirus pathology, suggesting an altered immune response and dampened viral control. However, no major alterations in T-cell phenotype in the aIFNpos patients could be identified. Furthermore, SARS-CoV-2–specific T-cell function was retained in the individuals assessed in this study.

In line with our observations on T cells, we found also the B-cell phenotype and BCR repertoire remained stable even in the presence of aIFN-Abs. Furthermore, when studying the levels of antibodies against SARS-CoV-2 and other viral infections, no major imprint was observed. These data are in line with recent reports showing that patients with aIFN-Abs were able to mount a sufficient Ab production upon vaccination [26, 27]. We conclude that despite an impaired type I IFN response, an efficient humoral response can be established against SARS-CoV-2 and other viruses, but that detected antibody levels might be uncoupled from Ab-mediated protection in patients with aIFN-Abs.

In review of our and other's data, it appears that the compromised IFN response in aIFNpos patients has a rather narrow impact and does not lead to a broad inhibition of peripheral immune responses. In this context, van der Wijst et al showed in single-cell RNA sequencing experiments that a hampered IFN response was common to critically ill COVID-19 patients, but aIFN-Abs associated with more pronounced alterations in the myeloid/DC compartment [7]. This is in line with our data, as we show that soluble factors and adaptive immune cell phenotypes are largely unaltered in aIFNpos patients but we did observe altered DC and T-cell numbers and skewed immune pathways. Therefore, we conclude that the impact of aIFN-Abs is more on the cellular than soluble immune compartment and possibly more on the innate than on the adaptive side of immunity.

IFNs are also of importance locally, at the site of infection [28–30]. In this context, the recent report on the presence of aIFN-Abs in bronchoalveolar lavage fluid in patients with severe COVID-19 is of interest [31], as it indicates that local effects are also perturbed. Thus, it might be that aIFN-Abs lead to an altered immune response in the tissue. It can be hypothesized that the local effect of aIFN-Abs leads to elevated influx of immune cells, resulting in the loss of circulating immune cells we and others observed, while long-term adaptive immunity remains unchanged. Future studies should investigate this in more detail, and in this context whether aIFN-Abs affect local antiviral responses.

This study has limitations. Due to the moderate prevalence of aIFN-Abs in critical COVID-19 patients, only a relatively small number of patients with aIFN-Abs could be identified. Furthermore, we did not assess neutralizing activity or subtype specificity of detected aIFN-Abs. Nor did we assess for the presence of aIFN-Abs for IFN-β or ω. However, most individuals harbor antibodies against several type I IFN subtypes and aIFN-Abs against IFN-α subtypes are more prevalent [6] and detected autoantibodies are mostly neutralizing [6, 7, 24]. Lastly, it was not possible to perform all analysis on all patients, either due to lack of biological sample or experimental issues such as low viability of cells.

In conclusion, our data suggest that aIFN-Abs can be stable over a long period of time and that their presence appears to be rather specific to certain infections such as COVID-19 caused by SARS-CoV-2. Furthermore, we conclude that these autoantibodies do not cause major alterations in the soluble immune compartment but do hamper cellular immunity, as evident by a loss within the T-cell and dendritic cell compartments. Future studies should investigate consequences of aIFN-Abs in the context of other respiratory infections and their local effect at the site of infection.

## Supplementary Data

Supplementary materials are available at *The Journal of Infectious Diseases* online (http://jid.oxfordjournals.org/). Supplementary materials consist of data provided by the author that are published to benefit the reader. The posted materials are not copyedited. The contents of all supplementary data are the sole responsibility of the authors. Questions or messages regarding errors should be addressed to the author.

## Supplementary Material

jiae036_Supplementary_Data

## Notes

***Financial support***. This work was supported by the European Research Council under the European Union Horizon 2020 Research and Innovation Program (grant number 948692); the Swedish Research Council; the Swedish Cancer Society; the Swedish Society for Medical Research; the Swedish Foundation for Strategic Research; Knut and Alice Wallenberg Foundation; the Novo Nordisk Foundation; the Center for Innovative Medicine at Karolinska Institutet; the South-Eastern Norway Regional Health Authority; Oslo University Hospital; Nordstjernan; Stockholms Läns Landsting; and Karolinska Institutet. Benedikt Strunz was supported by Stockholms Läns Landsting (clinical postdoctoral appointment).
