# Supplementary material for: Type I Interferon Autoantibodies Correlate With Cellular Immune Alterations in Severe COVID-19
Source: J Infect Dis. 2024 Feb 29;230(2):e318–26. doi: 10.1093/infdis/jiae036 (PMC11326830; doi:10.1093/infdis/jiae036)
Supplement: jiae036_Supplementary_Data [file jiae036_supplementary_data.zip › 240131_Supplementary materials_clean.docx]

**Supplementary Materials**

**Type I interferon auto-antibodies correlate with cellular immune alterations in severe COVID-19**

Benedikt Strunz, Christopher Maucourant, Adi Mehta, Hui Wan, Likun Du, Dan Sun, Puran Chen, Anna Nordlander, Yu Gao, Martin Cornillet, Jonna Bister, Egle Kvedaraite, Wanda Christ, Jonas Klingström, Daniel Geanon, Åsa Parke, Anna Ekwall-Larson, Laura Rivino, Paul A. MacAry, Soo Aleman, Marcus Buggert, Hans-Gustaf Ljunggren, Qiang Pan-Hammarström, Fridtjof Lund-Johansen, Kristoffer Strålin and Niklas K. Björkström on behalf of the Karolinska KI/K COVID-19 Study Group.

**Table of content**

- Supplementary methods
- Supplementary Figure 1
- Supplementary Figure 2
- Supplementary Figure 3
- Supplementary Figure 4
- Supplementary Figure 5
- Supplementary Table 1
- Supplementary Table 2

**Supplementary methods**

*Ethics statement*

The study of COVID-19 patients was approved by the Swedish Ethical Review Authority (2020-01558). Informed consent was obtained from the study participants. For patients treated at the ICU, the ethical authority approved sampling prior to consent. If an ICU treated study subject died prior to consent, the ethical authority approved that consent could be assumed. Separate ethical approvals covered cohorts of sepsis (2017/1358-1), hantavirus infected (2009/2083-31/2), yellow fever virus vaccinated (2017/1433-31/1) and dengue virus infected (DSRB 2013/00209 and DSRB 2008/00293) patients. All study subjects provided written informed consent.

*Sample acquisition*

Venous blood samples were collected in heparin tubes for determination of absolute immune cell counts as well as isolation of peripheral blood mononuclear cells (PBMCs) and in serum tubes for collection of serum. For the latter, after complete coagulation tubes were centrifuged and serum aliquoted and frozen at -80°C. Immune cell counts were determined from whole blood via BD Trucount Tubes (BD Biosciences) according to manufacturer’s instructions. PBMCs were isolated from heparinated blood via Ficoll gradient centrifugation. After separation, cells from the interphase were collected and washed twice. Cells were then counted using an automated cell counter (Countess II,ThermoFisher®) with trypan blue staining identifying dead cells and immediately frozen in FCS + 10% DMSO.

*Flow cytometric analysis*

For experimentation, PBMCs were thawed and 2*10^6 cells were stained in 96-well V-bottom plate (for antibodies, see Table S2A). Live/Dead cell discrimination was performed with fixable viability dye (Invitrogen). Cells were washed with FACS buffer before being acquired on a BD FACSymphony with 355-, 405-, 488-, 561-, and 640-nm lasers. FCS3.0 files were exported from the FACSDiva and imported into FlowJo v.10.6.2 for subsequent analysis (see supplemental methods for detailed flow cytometric analysis and assessment of T cell function).

*Flow cytometric assessment of T cell function*

Cryopreserved PBMCs were thawed quickly, resuspended in complete medium in the presence of DNase I (10 U/ml; Sigma-Aldrich), and rested at 1*10^6 cells/well in 96-well U-bottom plates for 3 hours at 37°C. Then, the media was supplemented with anti-CXCR5–BB515 (clone RF8B2; BD Biosciences) and unconjugated anti-CD40 (clone HB14; Miltenyi) followed 15 min later by the (Spike+nucleocapsid+membrane+envelope) peptide pool (0.5 μg/ml). Cells were then incubated at 37°C and 5% CO2 for 12 hours. After stimulation, cells were washed in FACS buffer. Cells were then stained with the antibodies detailed in table S2B. Briefly, cells were first stained for viability, then chemokine receptors at 37°C, followed by surface markers at room temperature. Cells were finally fixed with 1% paraformaldehyde in PBS and acquired using a FACSymphony A5 (BD Biosciences). Data were analyzed in FlowJo (version 10.6.2).

*Flow cytometric analysis*

The antibody mix was composed by titrated antibodies diluted in FACS buffer (PBS, 5mM EDTA, 1% FCS) supplemented with Brilliant stain Buffer (BD Biosciences) and samples stained for 15 minutes at room temperature for extracellular and 30 minutes for intracellular staining. Between the steps, cells were washed 3 times using FACS Buffer. For intracellular staining, cells were permeabilized with the Foxp3/Transcription Factor Staining Kit (eBioscience). The following plug-ins were used: DownSample (3.2), UMAP (3.1), and PhenoGraph (2.4). After compensation, single populations were gated for as displayed in supplementary Figures 3 and 4. For unsupervised analysis, events were first downsampled from the B cell or T cell gate across all samples using DownSample. One sample of an aIFNpos patient was excluded from T cell analysis due to too low cell recovery, and the matched aIFNneg sample was not used for UMAP comparison to avoid biased comparison. Status for Interferon antibodies was added as additional parameter and all samples concatenated for analysis. UMAP and Phenograph was performed on the parameters displayed in supplementary Figures 3 and 4. After Phenograph analysis, concatenated samples were deconvoluted and the relative abundance of the aIFNpos and aIFNneg groups was calculated for each cluster and the three most specific to either group investigated in more detail.

*BCR sequencing*

Total RNA was extracted from each PBMC sample and was used for BCR repertoire sequencing by commercially available RepSeq+ Cassettes (iRepertoire Inc., Huntsville, AL, USA). Library amplification was performed with commercial multiplex primers covering human immunoglobin genes from constant (C) region to variable (V) region. Amplified libraries were multiplexed and pooled for sequencing on the Illumina MiSeq platform using a 600-cycle kit (PE300). Sequencing raw data were preprocessed using MiGEC v1.2.9. Then, the merged clean reads were mapped to germline V, diversity (D), joining (J) and C reference sequences from IMGT database by MiXCR v3.0.14. BCR clonotype was defined by the same V gene, J gene, and CDR3 amino acid sequence. The number of unique molecular identifiers (UMIs) in each unique clonotype was considered as the clonotype size. Clonal diversity and clonal expansion were evaluated by the Shannon index and the Gini index, separately. The SHM rate was calculated as the number of mismatches in the V region divided by the alignment germline length.

*Serological analysis*

Antibodies to viral antigens and type I interferons were measured using a multiplexed bead-based assay [31]. The following antigens were used: SARS-CoV-2: Full-length spike protein [32], receptor-binding domain [33], Nucleocapsid produced in bacteria (Prospecbio, Israel) or in mammalian cells (gift from Jan Terje Andersen, Oslo, Norway), biotinylated peptide antigens corresponding to epitopes in Spike and nucleocapsid [34], HCOVs: S1 domains from OC43, HKU1, 229E and NL63 [35], full-length spike proteins from OC43 and NL63 and HA-antigen from OC43 (Sino biologicals), peptides from EBV EBNA1 and Rhinovirus A [34]. Hemagglutinin from Influenza H1N1 (2009) and H3N2 (1968) were obtained from Gunnveig Grødeland (Oslo University Hospital). Plasmids encoding GST-tagged type I interferons were obtained from the DNASU plasmid repository (Tempe, AZ, USA). Proteins were produced using an in vitro transcription and translation kit (ThermoFisher). Polymer beads with fluorescent barcodes were coupled successively to neutravidin (ThermoFisher) and viral antigens chemically biotinylated at 1:1 biotin to protein molar ratio with sulfo-LC-NHS-biotin (ProteoChem, USA). For binding of GST-tagged interferons, the beads were first coupled to biotinylated goat anti-GST (SICGEN, Portugal). The beads were pooled to generate bead-based arrays, which were kept at 4-8 ^o^C for up to 1 month or at -70^o^C for long term storage. Serum was diluted 1:100 or 1:1000 for measurement of antibodies to viral proteins or type I interferons, respectively. The PBS assay buffer contained 1% Tween 20, 10ug/ml D-biotin, 10 ug/ml Neutravidin and 0.1% Sodium Azide. Diluted serum was incubated with bead-based arrays in 384 well plates for 30 minutes at 22^o^C at constant agitation (anti-viral antibodies) or overnight at 4-8^o^C (anti-inferferons). Anti-interferon bead arrays were performed at 4-8^o^C overnight to enhance signal to noise ratio. Beads were next washed three times in PBS 1% Tween 20 (PBT), labelled with R-Phycoerythrin (R-PE)-conjugated goat anti-human IgG Fc or R-PE conjugated anti-human IgA (Jackson Immunoresearch) and analyzed by flow cytometry (Attune Next, Thermo). Flow cytometry data files (fcs 3.1) were analyzed in WinList. The median R-PE fluorescence intensity (MFI) of each bead subset was exported to Excel. The MFI of beads coupled with viral antigens was divided by that measured on beads coupled with neutravidin only (relative MFI, rMFI).

*Quantification and statistical analysis*

Statistical analysis was either performed in R v.4.0.5 and Graphpad Prism v8.3.0. Packages used in R were tidyr v.1.1.3, EnhancedVolcano v.1.8.0, factoextra v.1.0.7, reshape2 v.1.4.4, ggplot2 v.3.3.5, tidyverse 1.3.1. Fisher’s exact test was applied when calculating contingency tables for categorical variables. For comparison of aIFNpos and aIFNneg groups in the soluble proteome, a Welch’s t-test, to correct for unequal variance, with FDR-corrected p-values was applied for determining significant differences since NPX values represent log2 transformed data. In flow cytometric analysis of absolute cell counts, a Mann Whitney- test with FDR-adjusted p-values was applied for screening for differences. For comparison of three groups, a Kruskal-Wallis test followed by Dunn’s test for multiple comparisons was applied. Pathway analysis was conducted using QIAGEN's Ingenuity Pathway Analysis (IPA) software. The criteria for significance were set at an absolute Z-score threshold of 2 and a false discovery rate (FDR)-corrected *P*-value of 0.01.

**Supplementary Figures**

**Figure S1. (A)** Autoantibodies against indicaed type I Inteferons in patients with autoimmune polyendocrine syndrome (APS, n=2) that were included as positive control. **(B-D)** Volcano plots comparing the soluble proteome for the indicated groups of healthy individuals (n=18), aIFNneg patients (n=231), aIFNneg patients with critical COVID-19 (ICU, n=96), aIFNpos with critical COVID-19 (ICU, n=7) and aFNneg with severe COVID-19 (non-ICU, n=135), Welch’s t-test with FDR adjusted p-values was applied for determining significant differences. **(E)** Displayed are ISG-score, calculated from soluble proteome data based on 25 normalized proteins that are part of the Reactome *interferon signaling pathway*, and interferon lambda 1 (IFNL1) for the indicated groups. **(F)** Barplot displaying the ratio of mean counts for the indicated immune cell subset as determined via flow cytometry. Compared were aIFNpos or aIFNneg individuals with critical COVID-19 (n= 4 and 89, respectively), significant differences were calculated with Mann-Whitney Test, displayed in blue are p values >0.1, in yellow p<0.1 and red p<0.05. **(G)** Absolute counts of indicated immune cells in healthy controls (n=21) or aIFNneg (n=89) / aIFNpos (n=4) patients with critical COVID-19 (ICU patients).

**Figure S2. (A)** Exemplary plots for applied T cell gating strategy. **(B-E)** PhenoGraph and UMAP analysis of the T cell compartment. In short, positivity for aIFN-Abs was added as keyword and similar cell numbers exported and concatenated. After cleanup of outlier cells, UMAP and PhenoGraph analysis was performed on the markers displayed in **(E)**. **(B)** Deconvolution of aIFN-positivity and analysis in UMAP (left plot), overall sample distribution (middle plot) and total phonograph clusters (right plot) are displayed. **(C)** Relative contribution of aIFNneg and aIFNpos indviduals to each PhenoGraph cluster of which the three most enriched for the respective groups are displayed in (**D)**.

**Figure S3. (A** and **B),** Comparison of frequencies in identified subsets within the T cell (**A**) and B cell compartment (**B**). Displayed is the mean ratio of relative abundance between aIFNpos (n=7 for T cell and n=8 for B cell analysis, one sample removed due to low cell recovery) and aIFNneg (n=8 for B and T cell analysis) individuals. Differences between the groups were tested for with Mann-Whitney test, displayed are in blue p values >0.1, in yellow p<0.1 and in red p<0.05.

**Figure S4. (A)** Gating applied for identifying B cell subsets. **(B-E)** UMAP and PhenoGraph analysis of total B cells. Comparable cell numbers from aIFNneg and aIFNpos patient samples were downsampled and concatenated with status of aIFN as additional parameter. After a cleanup step to remove outlier cells UMAP and PhenoGraph were performed based on the in **(E)** displayed parameters. **(B)** Analysis of UMAP for aIFNpos and aIFNneg individuals (left plot), general distribution of included samples (middle plot) and calculated phonograph clusters (right plot). **(C)** Relative contribution of aIFNneg and aIFNneg patients to the indicated clusters. (**D)** display of the three clusters that were most enriched for either aIFNneg or aIFNpos individuals.

**Figure S5. (A and B)** Heatmap of antibody screening displaying the measured fluorescence for total IgG Fc against SARS-CoV2 specific proteins and peptides during acute COVID-19 (**A**, healthy n=18, aIFNneg n=225, aIFNpos n=9) or other pathogenic viruses (**B**, healthy n=18, aIFNneg n=241, aIFNpos n=10). **(C)** Median z-scores calculated from MFI values for total IgG Fc (left plot) and IgA (right plot) for the indicated viruses in the indicated groups. Samples were tested for significant differences among the respective groups with Kruskal Wallis test followed by Dunn’s test for multiple comparisons.

**Supplementary Table 1A**

|  | **aIFNneg** | **aIFNpos** |  |
| --- | --- | --- | --- |
| **Total** | 246 | 11* |  |
| **Sex (female, %)** | 58 (23.6%) | 3 (30%) | p=0.71 |
| **Age (years, median with range)** | 60 (18-99) | 64 (50-91) | p=0.34 |
| **BMI (kg/m2, median with range)** | 28 (17.9-55) | 25.3 (20.6-32.7) | p=0.23 |
| **Charlson Comorbitiy Index (median with range)** | 1 (0-9) | 1.5 (0-3) | p=0.78 |
| **Steroid treatment at hospital (%)** | 151 (61%) | 7 (70%) | p=0.75 |
| **Time symptom debut-first sampling (days, median with range)** | 15 (1-72) | 16.5 (9-33) | p=0.87 |
| **Peak CRP (mg/L, median with range)** | 298 (93-380) | 190 (11-620) | p=0.098 |
| **Peak Neutrophil count (x10^9/L, median with range)** | 9.3 (1.4 - 43) | 11.9 (8.2-40.6) | *p=0.038 |
| **Patients treated on ICU (%)** | 113 (45.9%) | 8 (80%) | p=0.0501 |
| **ICU patients: Time on ICU (days, median with range)** | 17 (1-76) | 19 (6-66) | p=0.51 |
| **-CU patients: Peak CRP (mg/L, median with range)** | 319 (42-620) | 298 (93-380) | p=0.60 |
| **ICU patients: Peak Neutrophil count (x10^9/L, median with range)** | 13.3 (5.9-43) | 15 (8.2-40.6) | p=0.54 |
|  |  |  |  |

*One patient has no further clinical data available

**Supplementary Table 1A. Patient characteristics of COVID-19 patients.** Displayed are clinical parameters either as median with range or as number of patients with percentage. Statistical differences were calculated either with Mann Whitney test or Fisher’s exact test for contingency tables.

**Table S1B**

| **Autoantibody positive (%)** | 1 (3%) |
| --- | --- |
| **Sex (female, %)** | 14 (38%) |
| **Age (years, median with range)** | 72 (44-100) |
| **Charlson Comorbitiy Index (median with range)** | 2 (0-10) |
| **SOFA score (median with range)** | 3 (0-7) |
| **Clinical diagnosis:** |  |
| **-Influenza** | 6 (16%) |
| **-Pneumonia (bacterial)** | 10 (27%) |
| **-Urinary tract infection** | 9 (24%) |
| **-Other** | 12 (32%) |

**Supplementary Table 1B. Patient characteristics of sepsis patients.** Clinical parameters of patients admitted to hospital with sepsis.

**Table S2A,** Antibodies and reagents used for T and B cell phenotyping

| **Fluorochromes** | **Marker** | **Company** | **Dilution** | **Clone** |
| --- | --- | --- | --- | --- |
| BUV805 | CD85J | BD Biosciences | 25 | GHI/75 |
| BUV737 | CD10 | BD Biosciences | 25 | HI10a |
| BUV661 | CD73 | BD Biosciences | 50 | AD2 |
| BUV615-P | CD 24 | BD Biosciences | 50 | ML5 |
| BUV563 | CD21 | BD Biosciences | 50 | B-LYN4 |
| BUV496 | CXCR5 | BD Biosciences | 50 | RF8B2 |
| BUV395 | CD 19 | BD Biosciences | 100 | SJ25-C1 |
| BV786 | CD 20 | Biolegend | 100 | 2H7 |
| BV750 | IgD | BD Biosciences | 50 | IA6-2 |
| BV711 | CD40 | BD Biosciences | 25 | 5c3 |
| BV650 | IGM | BD Bioscience | 50 | II/41 |
| BV605 | CXCR3 | BD Biosciences | 25 | 1C6 |
|  | DCM Yellow | Invitrogen | 400 |  |
| BV570 | CD3 | Biolegend | 50 | UCHT1 |
| BV570 | CD14 | Biolegend | 50 | M5E2 |
| BV510 | HLA-DR | Biolegend | 50 | L243 |
| BV421 | CD138 | Biolegend | 50 | MI15 |
| BB790-P | KI-67 | BD Biosciences | 50 | B56 |
| BB755-P | 45 RB | BD Biosciences | 50 | MT4 |
| BB700 | BaffR | BD Biosciences | 50 | 11C1 |
| Biotin | CD27 | eBioscience | 100 | O323 |
| BB515 | CD70 | Miltenyi | 50 | REA230 |
| BB630 | Streptavidin | BD Biosciences | 400 |  |
| PE-Cy7 | IgA | Miltenyi | 50 | IS11-8E10 |
| PE-Cy5.5 | CD11c | Life technologies | 200 | MHCD11c18 |
| PE-CY5 | IgG | BD Biosciences | 50 | G18-145 |
| PE-CF594 | T-bet | BD Biosciences | 100 | O4-46 |
| PE | CD95 | BD Biosciences | 50 | DX2 |
| APC-Cy7 | BCL-6 | BD Biosciences | 25 | K112-91 |
| Alexa700 | CD 38 | BD Biosciences | 100 | HIT2 |
| APC | FCRL-4 | BD Biosciences | 25 | A1 |
| BUV805 | CXCR5 | BD Biosciences | 25 | RF8B2 |
| BUV737 | CD69 | BD Bioscience | 25 | FN50 |
| BUV661 | CD38 | BD Biosciences | 25 | HIT2 |
| BUV615 | CD4 | BD Biosciences | 50 | SK3 |
| BUV563 | CD45RA | BD Biosciences | 25 | HI100 |
| Biotin | CD161 | BD Biosciences | 25 | DX12 |
| BUV395 | CD25 | BD Biosciences | 25 | 2A3 |
| BUV496 | Streptavidin | BD Biosciences | 400 |  |
| BV786 | HLA-DR | Biolegend | 50 | L243 |
| BV750 | CD3 | Biolegend | 100 | SK7 |
| BV711 | CCR6 | Biolegend | 25 | G034E3 |
| BV650 | CD95 | Biolegend | 50 | DX2 |
| BV605 | CXCR3 | BD Biosciences | 25 | 1C6 |
| BV570 | CD8 | Biolegend | 50 | RPA-T8 |
|  | DCM acqua | Invitrogen | 100 |  |
| BV421 | ICOS | BD Biosciences | 25 | DX29 |
| BB790-P | KI-67 | BD Biosciences | 50 | B56 |
| BB700 | CD7 | BD Biosciences | 50 | M-T701 |
| BB660-P | CD57 | BD Biosciences | 50 | nk-1 |
| BB630-P | CXCR6 | BD Biosciences | 25 | 13B 1E5 |
| BB515 | CCR10 | BD Biosciences | 50 | 1B5 |
| PE-Cy7 | CCR4 | BioLegend | 25 | L291H4 |
| PE-Cy5.5 | TCRgd | Beckman Coulter | 50 | IMMU510 |
| PE-CY5 | CXCR4 | Biolegend | 25 | 12G5 |
| PE-CF594 | CCR7 | BioLegend | 25 | G043H7 |
| PE | PD1 | BD Biosciences | 50 | EH12.1 |
| APC-Cy7 | TCRVA7.2 | BioLegend | 100 | 3C10 |
| Alexa700 | FOXP3 | Invitrogen | 25 | PCH101 |
| APC | Tox | Miltenyi | 25 | REA473 |

Table S2B, Protocol applied for staining of T cell function

| STEP 1: Stain for viability at room temperature for 10 minutes. | | | | |
| --- | --- | --- | --- | --- |
| **Marker** | **Fluorophore** | **Supplier** | **Dilution** | **Clone** |
| LIVE/DEAD fixable aqua | For 405 nm excitation | Invitrogen | 1X in PBS | - |
| STEP 2: Stain for chemokine receptors at 37°C for 10 minutes. | | | | |
| **Marker** | **Fluorophore** | **Supplier** | **Dilution** | **Clone** |
| CCR7 | APC-Cy7 | BioLegend | 1:50 | G043H7 |
| CCR4 | BB700 | BD | 1:50 | 1G1 |
| CCR6 | BUV737 | BD | 1:75 | 11A9 |
| CXCR3 | AF647 | BioLegend | 1:100 | G025H7 |
| STEP 3: Stain remaining antibodies at room temperature for 30 minutes in BD Brilliant Stain Buffer Plus. | | | | |
| **Marker** | **Fluorophore** | **Supplier** | **Dilution** | **Clone** |
| CD40L | BV421 | BioLegend | 1:25 | 24-31 |
| 4-1BB | PE-Cy7 | BioLegend | 1:25 | 4B4-1 |
| CD4 | BUV496 | BD | 1:25 | SK3 |
| CD14 | BV510 | BioLegend | 1:100 | M5E2 |
| CD19 | BV510 | BioLegend | 1:100 | HIB19 |
| CD45RA | BV570 | BioLegend | 1:200 | HI100 |
| CD69 | BV650 | BioLegend | 1:50 | FN50 |
| CD3 | BUV805 | BD | 1:50 | UCHT1 |
| CD8 | BUV395 | BD | 1:250 | RPA-T8 |
| HLADR | BV605 | BD | 1:33 | G46-6 |
| CD95 | PEDazzle594 | BioLegend | 1:50 | DX2 |
| PD1 | BUV615 | BioLegend | 1:50 | EH12.1 |
| CD127 | PE-Cy5 | BioLegend | 1:100 | A019D5 |
| Integrin B7 | PE | Biolegend | 1:50 | FIB504 |
| CD38 | APC-R700 | BD | 1:50 | HIT2 |
| CD71 | BUV771 | BD | 1:50 | M-A712 |
| CD103 | BV711 | Biolegend | 1:50 | Ber-ACT8 |
| STEP 4: Wash and fix cells in 1% paraformaldehyde. | | | | |
